# Gait parameters associated with hallux valgus: a systematic review

**DOI:** 10.1186/1757-1146-6-9

**Published:** 2013-03-12

**Authors:** Sheree E Nix, Bill T Vicenzino, Natalie J Collins, Michelle D Smith

**Affiliations:** 1 Division of Physiotherapy, School of Health and Rehabilitation Sciences, The University of Queensland, Brisbane, Australia; 2 School of Clinical Sciences, Queensland University of Technology, Kelvin Grove, Brisbane, Australia; 3 Department of Mechanical Engineering, Melbourne School of Engineering, The University of Melbourne, Melbourne, Australia; 4 Department of Physiotherapy, Melbourne School of Health Sciences, The University of Melbourne, Melbourne, Australia

## Abstract

**Background:**

Hallux valgus (HV) has been linked to functional disability and increased falls risk in older adults. However, specific gait alterations in individuals with HV are unclear. This systematic review investigated gait parameters associated with HV in otherwise healthy adults.

**Methods:**

Electronic databases (Medline, Embase, CINAHL) were searched to October 2011, including cross-sectional studies with clearly defined HV and non-HV comparison groups. Two investigators independently rated studies for methodological quality. Effect sizes (95% confidence intervals (CI)) were calculated as standardized mean differences (SMD) for continuous data and risk ratios (RR) for dichotomous data.

**Results:**

Nine studies included a total of 589 participants. Three plantar pressure studies reported increased hallux loading (SMD 0.56 to 1.78) and medial forefoot loading (SMD 0.62 to 1.21), while one study found reduced first metatarsal loading (SMD −0.61, CI −1.19 to −0.03) in HV participants. HV participants demonstrated less ankle and rearfoot motion during terminal stance (SMD −0.81 to −0.63) and increased intrinsic muscle activity (RR 1.6, 1.1 to 2.2). Most studies reported no differences in spatio-temporal parameters; however, one study found reduced speed (SMD −0.73, -1.25 to −0.20), step length (SMD −0.66 to −0.59) and less stable gait patterns (SMD −0.86 to −0.78) in older adults with HV.

**Conclusions:**

HV impacts on particular gait parameters, and further understanding of potentially modifiable factors is important for prevention and management of HV. Cause and effect relationships cannot be inferred from cross-sectional studies, thus prospective studies are warranted to elucidate the relationship between HV and functional disability.

## Background

Hallux valgus (HV) is a common foot deformity [1] that significantly impacts on self-reported function and quality of life [2-4], and has been shown to increase falls risk in elderly individuals [5-8]. One proposed link between HV and increased risk of falls is gait instability [9,10]. Considering the significant morbidity and mortality associated with falls [11,12] and the importance of maintaining a high level of function in older adults, understanding altered gait parameters in people with HV and their association with functional impairment is essential.

The first metatarsophalangeal joint (MTPJ) functions as a vital pivot for transfer of body weight during the late stance phase of gait [13-15]. It is plausible that progressive subluxation of the first MTPJ in HV [16] might interfere with efficient toe-off, and several studies have reported altered plantar pressures in individuals with HV, albeit with inconsistent findings for hallux loading [17,18] and forefoot loading [19-23]. Altered biomechanics such as first ray hypermobility [24] and excessive foot pronation are often proposed to be associated with the development of HV [25,26]. According to Perera et al. [26], kinematic parameters such as increased angle of gait, increased rearfoot eversion, reduced ankle dorsiflexion, and functional limitation of first MTPJ dorsiflexion may increase abductory ground reaction forces on the hallux during gait. Glasoe et al. [25] further describes the effect of excessive foot pronation on first MTPJ axis orientation. Muscle imbalance around the first MTPJ has also been noted in HV [27,28], which is important as the intrinsic muscles of the foot are key dynamic arch stabilisers [26,29]. Although each of these factors has been discussed, no systematic appraisal of existing literature investigating gait parameters in HV has been performed to date.

A rigorous and systematic synthesis of the literature is required, to make clear to both clinicians and researchers the current state of the evidence for gait function in individuals with HV, and to direct further endeavours in research and interventions for HV. The aim of this systematic review was to investigate gait parameters in otherwise healthy individuals with HV compared to controls.

## Methods

### Search strategy

Comprehensive searches of electronic databases (Medline, Embase, and CINAHL) were conducted by the first author for all years available up to October 2011, without language restriction. A highly sensitive search strategy was used and has previously been reported in detail [1]. Search terms included subject headings specific to each database, as well as keywords including “hallux valgus,” “bunion,” and “foot deformity” with truncation and proximity symbols. The search was limited by a second string of terms, including synonyms relating to cross-sectional, case–control or prospective study designs. Reference lists of relevant publications were hand-searched by the same investigator to retrieve all available studies.

### Inclusion criteria

Assessment of study eligibility was performed by one investigator. Titles and abstracts of all records identified by the search strategy were scanned for eligibility using the screening question: “Does the study discuss factors associated with HV?” Eligible full-text articles were then retrieved for detailed evaluation according to the following inclusion criteria: 1) clear definition of HV using angular criteria or categorical rating scale; 2) investigated association between HV and gait parameters; 3) study population of adults free of systemic disease; 4) cross-sectional or longitudinal study design with non-HV comparison group. Translations were obtained for articles published in languages other than English to determine their eligibility for inclusion. Authors were contacted for clarification of study methodology as required.

### Quality assessment and risk of bias

Included studies were assessed for methodological quality by two independent raters, with any disagreements remaining after a consensus meeting resolved by third party consultation. Title, journal, and author details were removed to de-identify articles prior to rating. Quality ratings were performed using the Epidemiological Appraisal Instrument (EAI) [30], which has been validated for assessment of observational studies. Thirty-one items from the original EAI were used, after removing items specifically relating to interventions, randomization, follow-up period, or loss to follow-up, that were not applicable to cross-sectional observational studies. Items were scored as “Yes” (score = 2), “Partial” (score = 1), “No” (score = 0), “Unable to determine” (score = 0), or “Not Applicable” (item removed from scoring). Scores for all applicable items were summed and an average score was determined, with a maximum possible score of 2 (range 0 to 2). To assess potential publication bias across included studies, visual inspection of funnel plots was conducted with effect sizes plotted against study quality scores, sample size and publication year.

### Data management

For all included studies, the following information was extracted by one investigator: publication details (author, year, publication type, country), sample characteristics (sampling frame, inclusion criteria, number of HV cases, number of control subjects, age, sex), and study methodology (study design, examiner details, definition of HV, associated factors investigated, reliability of measurement methods). In order to calculate effect sizes, means and standard deviations (SD) were recorded for HV and control participants for continuous variables, and raw counts for dichotomous variables. If a study reported data for subgroups of HV severity (e.g. mild and severe), these subgroups were combined for analysis by calculating a weighted average. Authors were contacted and requested to provide additional data where means and SDs were not provided in the original publication.

### Statistical methods

Pooling of data by meta-analysis was not performed due to lack of homogeneity of study methods and factors investigated. Where sufficient data was provided for continuous variables, standardized mean differences (SMD) were calculated as the difference between HV and control group means, divided by the pooled standard deviation. Interpretation of SMDs was based on previous guidelines [31]: small effect ≥ 0.2, medium effect ≥ 0.5, large effect ≥ 0.8. Where dichotomous data were reported, risk ratios (RR) were calculated as the number of participants with HV in the group with the associated factor present, divided by participants with HV in the group without the associated factor; thus, HV was considered the “event” for the purposes of calculating RR [32]. A RR of > 1.0 indicated that HV was more likely to be found in subjects with the associated factor present. Interpretation of RRs followed previous guidelines, with a small effect represented by RR ≥ 2.0, and a large effect represented by RR ≥ 4.0 [33]. Effect sizes were considered statistically significant if the 95% confidence interval (CI) did not contain zero for SMD or one for RR. Effect sizes and 95% CIs were calculated using Stata Version 10 (StataCorp LP, College Station, TX).

## Results

### Search results

**Figure 1:**
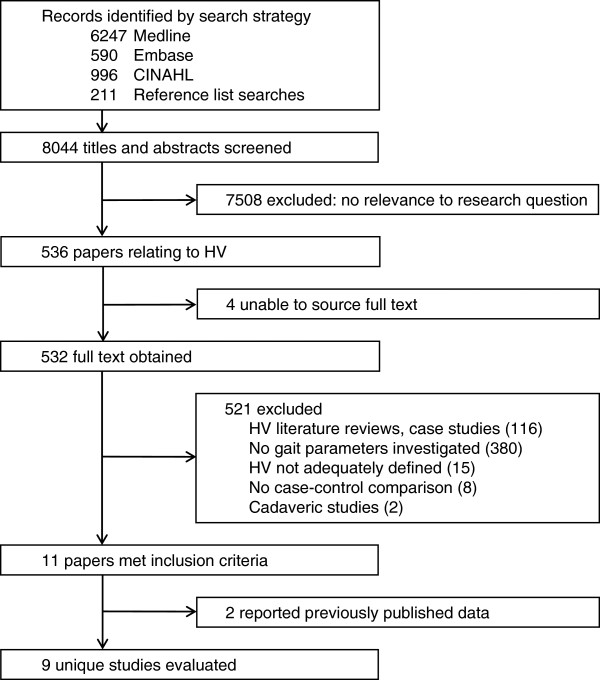
Flowchart of study selection procedure.

A total of 7833 records were retrieved by the electronic database search. Reference list searches identified another 211 potentially relevant titles. After screening all 8044 titles and abstracts, 532 full text articles relating to HV were examined. Translations were obtained for four studies relating to gait parameters that were published in languages other than English (1 Chinese, 1 Italian, 2 Japanese). Figure 1 outlines studies excluded at each stage of the selection process. After excluding literature reviews, case studies, cadaveric investigations, and studies that did not evaluate gait parameters (k = 498), 23 additional studies were excluded from analysis as they did not adequately define HV (k = 15) [19,20,23,34-45], did not include a control group (k = 4) [21,22,46,47] or did not perform comparison of HV subjects with controls in their analysis (k = 4) [18,48-50]. Comparison with a control group was required to answer the research question being addressed, and a clear definition of HV was essential to enable comparison between studies and to ensure validity of conclusions drawn from this review. Of the eleven studies meeting our inclusion criteria, two reported previously published data [51,52]; therefore, nine unique studies were evaluated [53-61].

### Quality assessment and risk of bias

Inter-rater agreement on the quality appraisal tool was 83.5% (46 disagreements out of 279 quality assessment items rated). Overall quality scores ranged from 0.26 to 1.19 out of a possible score range from 0 to 2 (Additional file 1). The majority of studies (7/9) [53-58,60] clearly reported their aims or objectives. All studies adequately reported sample characteristics (age, sex), and seven studies clearly reported subject inclusion criteria [53,54,56-60]. In contrast, no study provided an adequate description of the sampling frame, participation rate or non-responder characteristics. Reporting of statistical methodology was quite poor, with only three studies scoring “Yes” [56-58], and none providing details of sample size or power calculations. Basic data such as means and SDs were presented for all associated factors in six studies [53-57,60]; however, three studies reported insufficient data for some associated factors and therefore scored “Partial” [58,59,61]. Only two studies reported effect sizes that represented the magnitude or strength of association [56,60]. Three studies accounted for covariates such as sex and age, either by matching HV and control groups or statistical adjustment in their analyses [56-58]. All studies fulfilled our predetermined criterion for defining HV (using angular criteria or categorical rating scale); however, only four studies clearly described a quantitative measurement method for HV (a score of “Yes”) [53,55,56,60]. The remaining five studies provided incomplete details for measurement of HV angle, or used a scale that relied upon visual observation (a score of “Partial”) [54,57-59,61]. Measurement reliability was considered separately for assessment of HV and associated factors. Only three studies reported adequate reliability (coefficient > 0.7) for assessment of HV angle [53,57,60], and only two studies reported adequate reliability for all associated factors [53,60]. Another three studies [56-58] scored “Partial” for this item as reliability coefficients > 0.4 were reported, or the study made reference to measurement reliability documented in previous literature. Regarding potential publication bias, when SMDs for all associated factors were plotted against study quality scores, sample size and publication year, resulting funnel plots appeared symmetrical, indicating that publication bias was unlikely to have impacted findings from this review.

### Characteristics of included studies

Additional file 2 presents selected characteristics of the nine studies, including a total of 589 subjects (287 HV, 302 controls). Table 1 outlines age and sex characteristics of the study samples. In six included studies, the HV group comprised predominantly women [53-55,59-61], and one study only included female participants [56]. One study recruited a relatively even ratio of males to females (17:19) [58], while another study did not report male:female ratio [57]. Only one study matched HV and control groups by sex and age [58], although two studies demonstrated that the mean age of their HV and control groups were within three years [56,60]. Seven studies utilised a case–control study design [53-56,59-61], while two studies used cross-sectional designs and compared participants with moderate to severe HV to those with mild or no HV deformity [57,58]. The definition of HV varied between studies (see Additional file 2). Six studies used radiographic HV angle to define HV cases [53,55,56,59-61], two studies used the Manchester Scale [54,58], and the final study [57] reported visual observation using the following angular criteria: mild (HV angle <15°), moderate (HV angle 15-45°) or severe (HV angle >45°).

**Table 1:** Age (years)* and sex (male/female) characteristics of HV and control groups (9 studies)

**Study ID**	**Reference**		**HV group**	**Control group**	**Total**
Bryant 2000	[53]	N	30	30	60
		Sex	3/27	12/18	15/45
		Age	51.3 (range 28 to 74)	39.8 (range 23 to 68)	NR
Deschamps 2010	[54]	N	20	22	42
		Sex	4/16	9/13	13/29
		Age	47.4 (range 18 to 65)	37.5 (range 20 to 60)	NR
Kadono 2003	[55]	N	35 (57 feet)	18	53
		Sex	2/33	13/5	15/38
		Age	52.3 (range 12 to 77)	36 (range 22 to 68)	NR
Martinez-Nova 2010	[56]	N	79	98	177
		Sex	0/79	0/98	0/177
		Age	54.7±12.5	52.3±11.8	NR
Menz 2005	[57]	N	21	50	71
		Sex	NR	NR	24/47
		Age	NR	NR	80±4 (range 75 to 93)
Mickle 2011	[58]	N	36	36	72
		Sex	17/19	17/19	34/38
		Age	71.9±6.7	71.9±6.6	NR
Shimazaki 1981	[59]	N	28 (28 feet)	10 (10 feet)	38
		Sex	0/28	2/8	2/36
		Age	NR	NR	32 (range 20 to 65)
Taranto 2007	[60]	N	23 (36 feet)	20 (40 feet)	43 (76 feet)
		Sex	2/21	8/12	10/33
		Age	61.3±9.9 (range 45 to 79)	58.8±15.9 (range 28 to 82)	NR
Tokita 1991	[61]	N	15 (30 feet)	18 (36 feet)	33 (66 feet)
		Sex	1/14	4/14	5/28
		Age	40.7 (range 12 to 60)	26.8 (range 20 to 42)	NR

*Abbreviations:* HV, hallux valgus; NR, not reported.

* Age is reported as mean ± SD (range) unless details were not reported in original publication.

### Associated factors

Six studies investigated dynamic plantar pressure parameters [53,55,56,58,59,61], while five studies investigated spatio-temporal parameters, including angle of gait [54,56-58,60]. One study investigated gait stability, using accelerometers at the head and pelvis to measure the degree of rhythm of subject’s stride patterns [57], and another study investigated intersegmental joint kinematics throughout the gait cycle [54]. Only one included study investigated dynamic electromyographic (EMG) activity of the intrinsic foot muscles [59].

#### Plantar pressure

Table 2 presents SMDs and 95% CI for studies that investigated dynamic plantar loading variables. Effect sizes reveal significantly greater hallux peak pressure (SMD 0.56, 95% CI 0.05 to 1.08, n = 60) [53] and mean pressure (1.78, 1.43 to 2.13, n = 177) [56] in HV subjects compared to controls. One study (n = 60) [53] also found significantly greater peak pressure under the lesser toes in individuals with HV (0.56, 0.04 to 1.07). Effect sizes from two studies demonstrate that individuals with HV may have significantly greater peak pressure under the first metatarsal head (0.64, 0.12 to 1.16, n = 60 [53]; 0.70, 0.22 to 1.17, n = 72 [58]), and greater peak pressure under the second metatarsal head (1.21, 0.66 to 1.76, n = 60 [53]; 0.68, 0.21 to 1.16, n = 72 [58]). These studies also demonstrated greater pressure–time integral under the first metatarsal head (0.62, 0.14 to 1.09, n = 72 [58]) and significantly greater peak pressure under the third metatarsal head (0.99, 0.46 to 1.53, n = 60 [53]) in HV participants. Contrasting these findings, effect sizes from one study (n = 53) [55] showed HV participants to have significantly lower mean pressure under the first metatarsal when expressed as a percentage of total metatarsal pressure (−0.61, -1.19 to −0.03). Furthermore, one study (n = 33) [61] investigated centre of pressure pathways, with HV participants more likely to have a pathway terminating around the third metatarsal, rather than moving towards the first metatarsal and hallux throughout late stance phase as seen in control subjects (RR 2.3, 1.5 to 3.4). In contrast, several studies found no differences between HV and control participants. One study (n = 177) [56] found no differences between HV and controls in mean pressure under the first and second metatarsal heads, while several studies found no significant differences in hallux pressures [55,58,59], lesser toe pressures [55,56,58], pressure under the third, fourth and fifth metatarsal heads [55,56,58], and midfoot and heel peak pressure [53].

**Table 2:** Comparison of peak pressure, mean pressure, and pressure–time integral between HV and control groups (5 studies)

**Plantar pressure parameter**	**Study ID**	**Ref**	**N HV cases**	**N controls**	**SMD**	**95% CI**
*Hallux*						
Hallux peak pressure (N/cm2)	Bryant 2000	[53]	30	30	0.56*	**0.05 to 1.08**
Hallux peak pressure (kPa)	Mickle 2011	[58]	36	36	0.24	−0.23 to 0.70
Hallux mean pressure (% total toe pressure)	Kadono 2003	[55]	35	18	−0.37	−0.94 to 0.21
Hallux mean pressure (kPa)	Martinez-Nova 2010	[56]	79	98	1.78*	**1.43 to 2.13**
Hallux mean pressure (%BW)	Shimazaki 1981	[59]	28	10	0.37	−0.36 to 1.1
Hallux pressure–time integral (kPa*s)	Mickle 2011	[58]	36	36	−0.01	−0.47 to 0.45
*Lesser digits*						
D2 peak pressure (N/cm2)	Bryant 2000	[53]	30	30	−0.07	−0.58 to 0.43
D2 peak pressure (kPa)	Mickle 2011	[58]	36	36	0.36	−0.11 to 0.82
D3-5 peak pressure (N/cm2)	Bryant 2000	[53]	30	30	0.56*	**0.04 to 1.07**
D3-5 peak pressure (kPa)	Mickle 2011	[58]	36	36	−0.03	−0.49 to 0.43
D2 mean pressure (% total toe pressure)	Kadono 2003	[55]	35	18	0.57	−0.01 to 1.15
D3 mean pressure (% total toe pressure)	Kadono 2003	[55]	35	18	0.54	−0.04 to 1.11
D4 mean pressure (% total toe pressure)	Kadono 2003	[55]	35	18	−0.31	−0.88 to 0.27
D5 mean pressure (% total toe pressure)	Kadono 2003	[55]	35	18	−0.08	−0.65 to 0.49
D2-5 mean pressure (kPa)	Martinez-Nova 2010	[56]	79	98	0.02	−0.28 to 0.31
D2 pressure–time integral (kPa*s)	Mickle 2011	[58]	36	36	0.25	−0.22 to 0.71
D3-5 pressure–time integral (kPa*s)	Mickle 2011	[58]	36	36	−0.08	−0.55 to 0.38
*Metatarsal heads*						
M1 peak pressure (N/cm2)	Bryant 2000	[53]	30	30	0.64*	**0.12 to 1.16**
M1 peak pressure (kPa)	Mickle 2011	[58]	36	36	0.70*	**0.22 to 1.17**
M1 mean pressure (% total M pressure)	Kadono 2003	[55]	35	18	−0.61*	**−1.19 to −0.03**
M1 mean pressure (kPa)	Martinez-Nova 2010	[56]	79	98	0.14	−0.16 to 0.44
M1 pressure–time integral (kPa*s)	Mickle 2011	[58]	36	36	0.62*	**0.14 to 1.09**
M2 peak pressure (N/cm2)	Bryant 2000	[53]	30	30	1.21*	**0.66 to 1.76**
M2 peak pressure (kPa)	Mickle 2011	[58]	36	36	0.68*	**0.21 to 1.16**
M2 mean pressure (% total M pressure)	Kadono 2003	[55]	35	18	0.10	−0.46 to 0.67
M2 mean pressure (kPa)	Martinez-Nova 2010	[56]	79	98	0.07	−0.23 to 0.37
M2 pressure–time integral (kPa*s)	Mickle 2011	[58]	36	36	0.37	−0.10 to 0.84
M3 peak pressure (N/cm2)	Bryant 2000	[53]	30	30	0.99*	**0.46 to 1.53**
M3 peak pressure (kPa)	Mickle 2011	[58]	36	36	0.47	0.0 to 0.93
M3 mean pressure (% total M pressure)	Kadono 2003	[55]	35	18	0.32	−0.25 to 0.9
M3 mean pressure (kPa)	Martinez-Nova 2010	[56]	79	98	−0.06	−0.36 to 0.24
M3 pressure–time integral (kPa*s)	Mickle 2011	[58]	36	36	0.18	−0.28 to 0.65
M4 peak pressure (N/cm2)	Bryant 2000	[53]	30	30	0.29	−0.22 to 0.8
M4 peak pressure (kPa)	Mickle 2011	[58]	36	36	0.04	−0.42 to 0.50
M4 mean pressure (% total M pressure)	Kadono 2003	[55]	35	18	−0.23	−0.8 to 0.34
M4 mean pressure (kPa)	Martinez-Nova 2010	[56]	79	98	−0.14	−0.44 to 0.16
M4 pressure–time integral (kPa*s)	Mickle 2011	[58]	36	36	−0.18	−0.64 to 0.29
M5 peak pressure (N/cm2)	Bryant 2000	[53]	30	30	−0.04	−0.54 to 0.467
M5 peak pressure (kPa)	Mickle 2011	[58]	36	36	0.30	−0.17 to 0.76
M5 mean pressure (% total M pressure)	Kadono 2003	[55]	35	18	0.21	−0.37 to 0.78
M5 mean pressure (kPa)	Martinez-Nova 2010	[56]	79	98	−0.08	−0.38 to 0.22
M5 pressure–time integral (kPa*s)	Mickle 2011	[58]	36	36	0.11	−0.35 to 0.58
*Midfoot*						
Midfoot peak pressure (N/cm2)	Bryant 2000	[53]	30	30	0.06	−0.45 to 0.57
*Heel*						
Heel peak pressure (N/cm2)	Bryant 2000	[53]	30	30	−0.06	−0.57 to 0.45

*Abbreviations:* HV, hallux valgus; SMD, standardised mean difference; CI, confidence interval, M = metatarsal, D = digit.

* Indicates a statistically significant difference.

#### Spatio-temporal parameters

Table 3 presents data pertaining to spatio-temporal gait parameters. Of the four studies [54,56-58] that investigated these parameters, only one study (n = 71) [57] demonstrated significant differences between HV subjects and controls. Effect sizes show HV participants to have slower walking speeds on an irregular surface (SMD −0.73, -1.25 to −0.20), as well as a shorter average step length on a level surface (−0.66, -1.18 to −0.14) and irregular surface (−0.59, -1.11 to −0.07) [57]. Findings of another study (n = 72) were in contrast to this, with effect sizes showing no significant differences in comfortable walking speed or stride length between HV and controls [58]. There were also no significant differences found between HV subjects and controls in gait cycle duration, stance or swing phase duration, cadence or angle of gait parameters.

**Table 3:** Comparison of spatio-temporal parameters between HV and control groups (5 studies)

	**Study ID**	**Ref**	**N HV cases**	**N Controls**	**SMD**	**95% CI**
Gait cycle duration (s)	Deschamps 2010	[54]	20	22	−0.12	−0.72 to 0.49
	Martinez-Nova 2010	[56]	79	98	0.02	−0.28 to 0.31
Stance duration (% GC)	Deschamps 2010	[54]	20	22	0.05	−0.56 to 0.65
	Mickle 2011	[58]	36	36	0.05	−0.41 to 0.51
Swing duration (% GC)	Deschamps 2010	[54]	20	22	−0.05	−0.65 to 0.56
	Mickle 2011	[58]	36	36	−0.05	−0.51 to 0.41
Double support (% GC)	Mickle 2011	[58]	36	36	0.12	−0.35 to 0.58
Cadence (steps/min)						
Level surface	Martinez-Nova 2010	[56]	79	98	0.30	0.00 to 0.60
Level surface	Menz 2005	[57]	21	50	−0.09	−0.60 to 0.42
Irregular surface	Menz 2005	[57]	21	50	−0.36	−0.87 to 0.16
Comfortable walking speed (m/s)						
Level surface	Mickle 2011	[58]	36	36	0.17	−0.29 to 0.63
Level surface	Menz 2005	[57]	21	50	−0.50	−1.02 to 0.02
Irregular surface	Menz 2005	[57]	21	50	−0.73*	**−1.25 to −0.20**
Speed variability (cm/s)	Mickle 2011	[58]	36	36	0.24	−0.22 to 0.71
Average step length (cm)						
Level surface	Mickle 2011	[58]	36	36	0	−0.46 to 0.46
Level surface	Menz 2005	[57]	21	50	−0.66*	**−1.18 to −0.14**
Irregular surface	Menz 2005	[57]	21	50	−0.59*	**−1.11 to −0.07**
Step length variability	Mickle 2011	[58]	36	36	0.19	−0.27 to 0.65
Stride length (cm)	Mickle 2011	[58]	36	36	0	−0.46 to 0.46
Stride length variability (cm)	Mickle 2011	[58]	36	36	0.13	−0.33 to 0.6
Step width (cm)	Mickle 2011	[58]	36	36	0.27	0.19 to 0.74
Step width variability (cm)	Mickle 2011	[58]	36	36	−0.13	−0.59 to 0.34
Toe out angle (°)	Mickle 2011	[58]	36	36	0.26	−0.2 to 0.73
Left feet	Taranto 2007	[60]	18	20	0.29	−0.35 to 0.93
Right feet	Taranto 2007	[60]	18	20	0.49	−0.16 to 1.14

*Abbreviations:* HV, hallux valgus; SMD, standardised mean difference; CI, confidence interval.

* Indicates a statistically significant difference.

#### Harmonic ratio

With regard to stability of gait patterns in elderly individuals (Table 4), effect sizes from one study (n = 71) [57] showed that HV participants walking on an irregular surface had significantly lower harmonic ratios in the vertical plane, measured using accelerometry at the pelvis (SMD −0.78, -1.3 to −0.25) and head (−0.86, -1.39 to −0.33). The harmonic ratio indicates the degree of rhythm of linear acceleration during gait, with a lower ratio being indicative of a less stable gait pattern. No significant differences were found between groups when walking on a regular surface.

**Table 4:** Comparison of gait kinematics (1 study) and harmonic ratio (1 study) between HV and control groups

	**Study ID**	**Ref**	**N HV cases**	**N Controls**	**SMD**	**95% CI**
Harmonic ratio						
Pelvic V - level surface	Menz 2005	[57]	21	50	−0.41	−0.92 to 0.11
Pelvic V - irregular surface	Menz 2005	[57]	21	50	−0.78*	**−1.3 to −0.25**
Pelvic AP - level surface	Menz 2005	[57]	21	50	0.00	−0.51 to 0.51
Pelvic AP - irregular surface	Menz 2005	[57]	21	50	−0.10	−0.61 to 0.41
Pelvic ML - level surface	Menz 2005	[57]	21	50	0.00	−0.51 to 0.51
Pelvic ML - irregular surface	Menz 2005	[57]	21	50	−0.21	−0.72 to 0.30
Head V - level surface	Menz 2005	[57]	21	50	−0.41	−0.92 to 0.11
Head V - irregular surface	Menz 2005	[57]	21	50	−0.86*	**−1.39 to −0.33**
Head AP - level surface	Menz 2005	[57]	21	50	−0.24	−0.75 to 0.27
Head AP - irregular surface	Menz 2005	[57]	21	50	−0.18	−0.69 to 0.33
Head ML - level surface	Menz 2005	[57]	21	50	−0.21	−0.72 to 0.30
Head ML - irregular surface	Menz 2005	[57]	21	50	0.24	−0.27 to 0.76
Relative intersegmental joint motion (°)						
Hallux-Forefoot DF/PF (terminal stance)	Deschamps 2010	[54]	20	22	0.60	−0.02 to 1.22
Hallux-Forefoot DF/PF (terminal swing)	Deschamps 2010	[54]	20	22	0.70*	**0.08 to 1.33**
Forefoot-Hindfoot DF/PF (mid-swing)	Deschamps 2010	[54]	20	22	0.72*	**0.09 to 1.34**
Forefoot-Hindfoot AD/AB (mid-swing)	Deschamps 2010	[54]	20	22	0.58	−0.04 to 1.20
Hindfoot-Tibia INV/EV (mid-stance)	Deschamps 2010	[54]	20	22	−0.59	−1.21 to 0.03
Hindfoot-Tibia INV/EV (pre-swing)	Deschamps 2010	[54]	20	22	−0.60	−1.22 to 0.02
Hindfoot-Tibia INT/EXT (terminal stance)	Deschamps 2010	[54]	20	22	−0.63*	**−1.25 to −0.01**
Forefoot-Tibia DF/PF (terminal stance)	Deschamps 2010	[54]	20	22	−0.81*	**−1.44 to −0.18**

*Abbreviations:* HV, hallux valgus; SMD, standardised mean difference; CI, confidence interval; V, vertical; AP, anteroposterior; ML, mediolateral; DF, dorsiflexion; PF, plantarflexion; AD, adduction; AB abduction; INV, inversion; EV, eversion; INT, internal rotation; EXT, external rotation.

* Indicates a statistically significant difference.

#### Joint kinematics

One study (n = 42) [54] investigated lower limb kinematics throughout the gait cycle in HV participants compared to controls. Due to the complexity of this data, the authors reported intersegmental motion (means and SDs) only for phases of gait that showed a significant difference between groups, and these are listed in Table 4. The original publication included graphs representing mean intersegmental angles throughout the entire gait cycle; this graphical data was not examined further in this review. When effect sizes were calculated for the current review, only four kinematic parameters were found to be statistically significant (Table 4). During swing phase, HV participants demonstrated greater hallux dorsiflexion throughout terminal swing (SMD 0.70, 0.08 to 1.33) and greater dorsiflexion of the forefoot with respect to the hindfoot during mid-swing (0.72, 0.09 to 1.34). During terminal stance, the HV group showed less internal rotation of the hindfoot with respect to the tibia (−0.63, -1.25 to −0.01) and reduced forefoot-tibia dorsiflexion motion (−0.81, -1.44 to −0.18).

#### Muscle activity

Using fine-wire electrodes inserted into abductor hallucis, adductor hallucis, flexor hallucis brevis, and extensor hallucis brevis, one study (n = 38) [59] investigated EMG activity during the stance phase of gait. Participants were classified as having one of the following patterns of intrinsic muscle activity: 1) onset of abductor hallucis activity at heel strike, followed by activation of adductor hallucis, flexor hallucis brevis, then extensor hallucis brevis; or 2) simultaneous onset of all four intrinsic muscles at heel strike. The RR of those with HV having this pattern of simultaneous muscle activity was 1.6 (CI 1.1 to 2.2). This represents a small but statistically significant effect, and indicates that HV participants were more likely to exhibit early onset of intrinsic muscle activity. Furthermore, graphical data presented by these authors showed that HV participants had higher intrinsic muscle activity expressed as a percentage of peak muscle activation during stance. SMDs could not be calculated for this data as means and SDs were unavailable from the author.

## Discussion

Findings of this systematic review indicate that individuals with HV differ to healthy controls on particular gait parameters. Reduced ankle dorsiflexion and less rearfoot supination during terminal stance have been observed in individuals with HV. Early onset of intrinsic muscle activity at heel strike may occur in those with HV compared to controls. Patterns of altered loading under the hallux and medial metatarsal heads are apparent, although studies report inconsistent findings. Older individuals with moderate to severe HV may exhibit slower, less stable gait patterns with a shorter stride length, especially when walking on irregular surfaces. However, other basic spatio-temporal parameters including angle of gait show no significant differences between those with and without HV.

### Joint kinematics

While limited kinematic data are available, Deschamps et al. [54] showed that individuals with HV (n = 20) displayed reduced ankle dorsiflexion during terminal stance compared to controls (n = 22). This observation supports the concept that restricted ankle dorsiflexion may contribute to HV development via an early and increased forefoot loading. There may also be a tendency to compensate by externally rotating the foot, subsequently increasing valgus forces on the hallux [26]. Deschamps et al. [54] also reported less internal rotation of the rearfoot with respect to the tibia during terminal stance, which suggests less rearfoot supination during terminal stance in those with HV compared to controls. This is consistent with the suggestion that late stance phase pronation may contribute to the development of HV via disruption to first ray mechanics [26]. The terminal stance phase is of particular clinical relevance in HV, as this is when the highest ground reaction forces are exerted on the forefoot, and altered alignment of joint axes and moments may lead to pathology [25,62]. Another kinematic study was excluded from our analysis due to an inadequate definition of HV [35], which may have affected the validity of their study findings. However, it should be noted that Canseco et al. [35] found no significant differences between groups in hindfoot position throughout the gait cycle, although significantly reduced forefoot and hindfoot ranges of motion during certain phases of gait were noted in those with symptomatic HV (n = 33) compared to controls (n = 25). Further studies are warranted to investigate kinematic parameters in HV, particularly kinematics of the first ray [46] and first MTPJ during toe-off [62], as improvements in technology and foot modelling overcome some of the challenges involved with kinematic analysis of the foot.

### Muscle activity

An important finding of this systematic review is the lack of evidence regarding dynamic muscle function in HV, with only one included study having investigated muscle activity during gait [59]. The main findings of this study showed that individuals with HV had earlier onset of intrinsic muscle activity at heel strike. Abductor hallucis is known to have an important role in supporting the medial longitudinal arch [29,63], and early activation of intrinsic muscles may be an attempt to stabilise a hypermobile first ray. A further study by Hoffmeyer et al. [39] found abnormal muscle biopsies (abductor hallucis or first dorsal interosseous) in 53 out of 57 HV patients undergoing surgery, as well as abnormal surface EMG recordings during gait in HV patients (n = 19) compared to controls (n = 19); however, this study did not clearly define HV and was therefore excluded from our analysis. There are a number of obvious difficulties associated with recording EMG of intrinsic foot muscles dynamically, including the potential for cross-talk if using surface electrodes, and gait pattern alterations due to discomfort if using fine wire electrodes. It is also difficult to normalise EMG data in populations with HV due to reduced intrinsic muscle strength and the inability of participants to perform meaningful maximum voluntary contractions. Two previous EMG studies [27,28] have utilised static standing tasks and isometric contractions to quantify muscle imbalance in HV. Both studies found that abductor hallucis became less active in abduction of the hallux and more active in flexion during isometric tasks; however, muscle activity during gait was not evaluated. From the limited data available, it appears that muscle imbalance may be a significant factor in HV. Further studies are warranted to investigate how the timing and magnitude of muscle activity during gait may differ in those with HV compared to controls, particularly muscles related to function of the medial longitudinal arch and first ray (e.g. tibialis posterior, peroneus longus and intrinsic foot muscles). A better understanding of impaired muscle function in HV could guide clinical interventions aimed at retraining muscle activation patterns.

### Plantar pressures

Findings of plantar pressure studies to date are not in agreement regarding forefoot loading in HV. While effect sizes for two studies [53,58] demonstrate increased pressure under the medial forefoot in those with HV, Komeda et al. [64] report significantly lower first metatarsal pressures. Similarly, while two studies in this review reported increased pressure under the hallux in those with HV [53,56], previous reports have shown an inverse correlation between hallux plantar loading and increasing HV severity [17,18,38]. It should also be noted that some studies found no significant differences in hallux [58] and medial forefoot pressures [56] in HV subjects compared to controls.

Several considerations may help explain these inconsistent study findings. It is plausible that different plantar loading patterns may be found in different stages of HV progression, as soft tissues adapt to forefoot deformity and joint degeneration may develop in the first MTPJ [16]. The presence of foot pain may also lead to inconsistent plantar pressure findings in HV [65], as individuals with first MTPJ pain may adopt strategies to offload the painful area during gait. This tendency to adopt a more cautious or antalgic gait pattern secondary to painful foot deformity has been previously discussed by Crosbie et al. [66]. Although HV subjects were not compared with controls, Morag and Cavanagh [49] discussed several other structural and functional factors influencing loading under the first metatarsal and hallux, including hallux and first metatarsal length, range of motion at the ankle and first MTPJ, and sesamoid height. Future studies investigating plantar pressures in HV should consider severity of deformity, as well as presence of foot pain and other structural factors that may influence plantar pressures.

Another consideration when comparing results between plantar pressure studies is the different systems used for data collection and analysis. Two studies included in this review used the EMED system (Novel, Germany) [53,58], while other systems included Biofoot (IBV, Valencia, Spain) [56] and F-Scan (Tekscan, South Boston) [55] in-shoe systems, as well as older methods [59,61]. These systems each use different sensor technologies with varying sensor sizes and responsiveness, which may impact on results [67]. Furthermore, in-shoe plantar pressure analysis results will vary compared to barefoot pressure analysis due to the influence of footwear [67].

### Spatio-temporal parameters

Regarding spatio-temporal parameters, angle of gait, or toe-out angle, was not significantly different between HV and control subjects in the two studies that investigated this [58,60]. These studies provide no evidence to support the proposition that an abducted angle of gait contributes to the development of HV by increasing abduction forces on the hallux during propulsion [25]. However, to date no prospective studies have investigated this parameter and future studies could consider angle of gait due to its theoretical link with HV development. Several studies have shown that basic spatio-temporal parameters do not differ significantly between those with and without HV [54,56-58,60]. However, one study by Menz and Lord [57] found that older adults with moderate to severe HV walked slower along an irregular surface, had a reduced stride length when walking along level and irregular surfaces (Table 3), and demonstrated less stable gait patterns when walking along an irregular surface (Table 4) compared to controls. Therefore, while basic spatio-temporal parameters appear to be largely unaffected in individuals with HV, these parameters may be affected in older individuals with moderate to severe HV, especially during more challenging walking tasks such as on uneven surfaces.

### Clinical implications

Based on these findings, conservative interventions that target biomechanical foot function during gait are warranted in HV management. Such interventions may include orthoses designed to alter rearfoot motion and assist with efficient forefoot loading and toe-off. Retraining of muscle activitation patterns may be relevant, particularly targeting muscles that dynamically support the medial longitudinal arch. Stretching of the gastrocnemius-soleus complex and manual therapies aimed at improving talo-crural joint motion may facilitate increased ankle dorsiflexion during terminal stance. Clinical trials are needed to investigate the effects of such conservative interventions on gait parameters in populations with HV.

### Methodological considerations

Studies included in this review were somewhat limited in number, as stringent inclusion criteria were used to ensure the validity of overall conclusions drawn. The number of gait studies excluded for methodological reasons highlights the need for future studies to use rigorous study methodology. However, gait parameters investigated by the excluded studies were similar to those reported by included studies, with the majority of excluded studies investigating plantar pressures (k = 22) [18-23,34,36-50]. As discussed, one excluded study investigated three-dimensional segmental kinematics [35], and one study investigated muscle activity during gait using surface EMG [39]. Since the parameters investigated by excluded studies are similar to those reported by included studies, the authors believe that our systematic review thoroughly summarises the best available evidence regarding gait factors associated with HV.

Our quality assessment revealed several limitations of the available literature investigating gait parameters in HV. First, there was poor reporting of study recruitment methods, making it difficult to assess the generalisability of study results. Furthermore, no studies reported sample size calculations, meaning that null findings might be due to a lack of statistical power required to detect significant differences between groups. The importance of an adequate definition of HV has been discussed previously [1], and was therefore a predetermined inclusion criteria for this review. Differences in HV definition still existed between included studies and may have contributed to inconsistent study findings. Future studies should use a validated approach to HV assessment and diagnosis, such as the Manchester Scale [68] or measurement of HV angle using digital photographs or radiographs [69]. Another pertinent issue is the lack of age and sex-matching of HV and control groups, with only three studies adequately adjusting for both age and sex. Although peak pressures have been shown not to differ between men and women [70], gait parameters including plantar pressures vary significantly with age [50]. Finally, due to the cross-sectional designs utilised by these studies, causal relationships cannot be inferred from this data. Until prospective studies can be conducted in this area, the level of evidence for altered gait parameters in HV should be considered low, and results interpreted with appropriate caution. However, inherent difficulty exists in conducting a prospective study with sufficient follow-up to investigate a slowly progressive deformity such as HV.

## Conclusions

HV appears to have a significant impact on particular gait parameters. At heel strike individuals with HV demonstrate early onset of intrinsic muscle activity, and ankle dorsiflexion and rearfoot supination both appear to be reduced during terminal stance. Altered forefoot loading has also been reported, although results are inconsistent between studies. Elderly individuals with HV may exhibit less stable gait patterns, and reduced velocity and stride length when walking on an irregular surface; however, basic spatio-temporal parameters do not appear to be altered in HV. Methodological limitations of previous research have been discussed, highlighting the importance of clearly defining HV. It is also important to match HV and control participants for age and sex, or statistically adjust for these and other factors that may influence gait parameters, such as presence of foot pain. Although cross-sectional study designs prevent conclusions from being drawn regarding causality, the identification of gait parameters that may increase the risk of HV development is important for prevention and management. Interventions that target biomechanical foot function, such as muscle retraining, manual therapies and foot orthoses, may have the potential to prevent the progression of HV deformity and symptoms, and improve clinical outcomes. Finally, prospective studies would improve our understanding of how HV leads to functional disability and increased falls risk in older adults.

## Competing interests

The authors declare that they have no competing interests.

## Supplementary Material

Additional file 1 Results from quality assessment using the Epidemiological Appraisal Instrument (9 included studies). Click here for file

Additional file 2 Selected characteristics of included studies. Click here for file
